# ALS Variants of Annexin A11’s Proline-Rich Domain Impair Its S100A6-Mediated Fibril Dissolution

**DOI:** 10.1021/acschemneuro.3c00169

**Published:** 2023-07-11

**Authors:** Aman Shihora, Ruben D. Elias, John A. Hammond, Rodolfo Ghirlando, Lalit Deshmukh

**Affiliations:** † Department of Chemistry and Biochemistry, University of California San Diego, La Jolla, California 92093, United States; ‡ Scripps Research Biophysics and Biochemistry Core, The Scripps Research Institute, La Jolla, California 92037, United States; ∥ Laboratory of Molecular Biology, National Institute of Diabetes and Digestive and Kidney Diseases, National Institutes of Health, Bethesda, Maryland 20892, United States

**Keywords:** Intrinsically disordered regions, amyloids, neurodegenerative diseases, NMR, SPR, phase separation

## Abstract

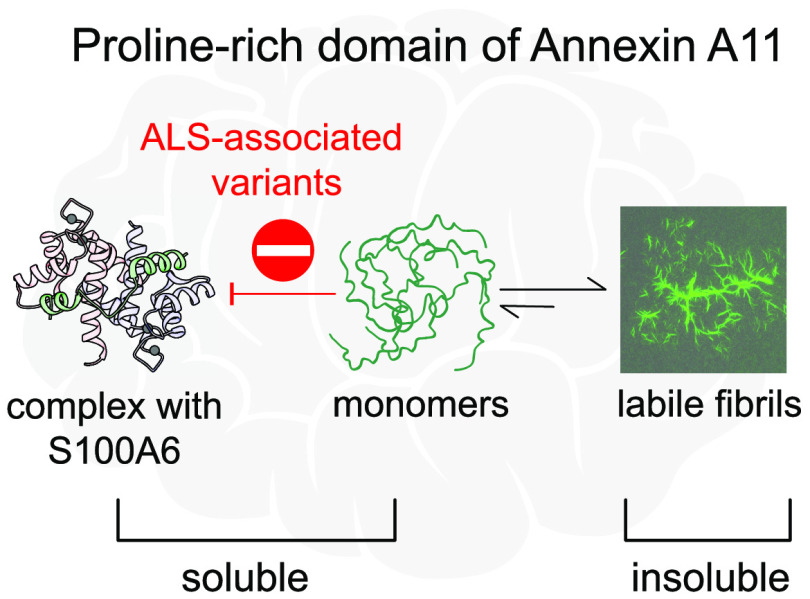

Mutations in the proline-rich domain (PRD) of annexin A11 are linked to amyotrophic lateral sclerosis (ALS), a fatal neurodegenerative disease, and generate abundant neuronal A11 inclusions by an unknown mechanism. Here, we demonstrate that recombinant A11-PRD and its ALS-associated variants form liquidlike condensates that transform into β-sheet–rich amyloid fibrils. Surprisingly, these fibrils dissolved in the presence of S100A6, an A11 binding partner overexpressed in ALS. The ALS variants of A11-PRD showed longer fibrillization half-times and slower dissolution, even though their binding affinities for S100A6 were not significantly affected. These findings indicate a slower fibril-to-monomer exchange for these ALS variants, resulting in a decreased level of S100A6-mediated fibril dissolution. These ALS-A11 variants are thus more likely to remain aggregated despite their slower fibrillization.

Annexins, calcium-dependent phospholipid-binding proteins, harbor multiple copies of a conserved core called an annexin repeat and a variable head domain.^1^ Among the 12 human annexins, annexin A11 comprises the largest head domain (196 residues; Figure 1A). This proline-rich domain (PRD, ∼30% prolines; Figure 1B) orchestrates A11’s phase separation, which is critical for its role in RNA granule transport.^2^ A11-PRD binds to S100A6 (calcyclin), a dimeric calcium-binding protein, and these interactions regulate the cell cycle.^3−5^ Four missense point mutations in A11-PRD, namely, G38R, D40G, G175R, and G189E, are linked to amyotrophic lateral sclerosis (ALS), a fatal neurodegenerative disease.^6^ Despite its importance, very little is known about A11-PRD. Here, we investigated its aggregation properties and interactions with S100A6. Our results elucidate how ALS-associated variants may promote neuronal A11 inclusions observed in ALS patients.

**Figure 1:**
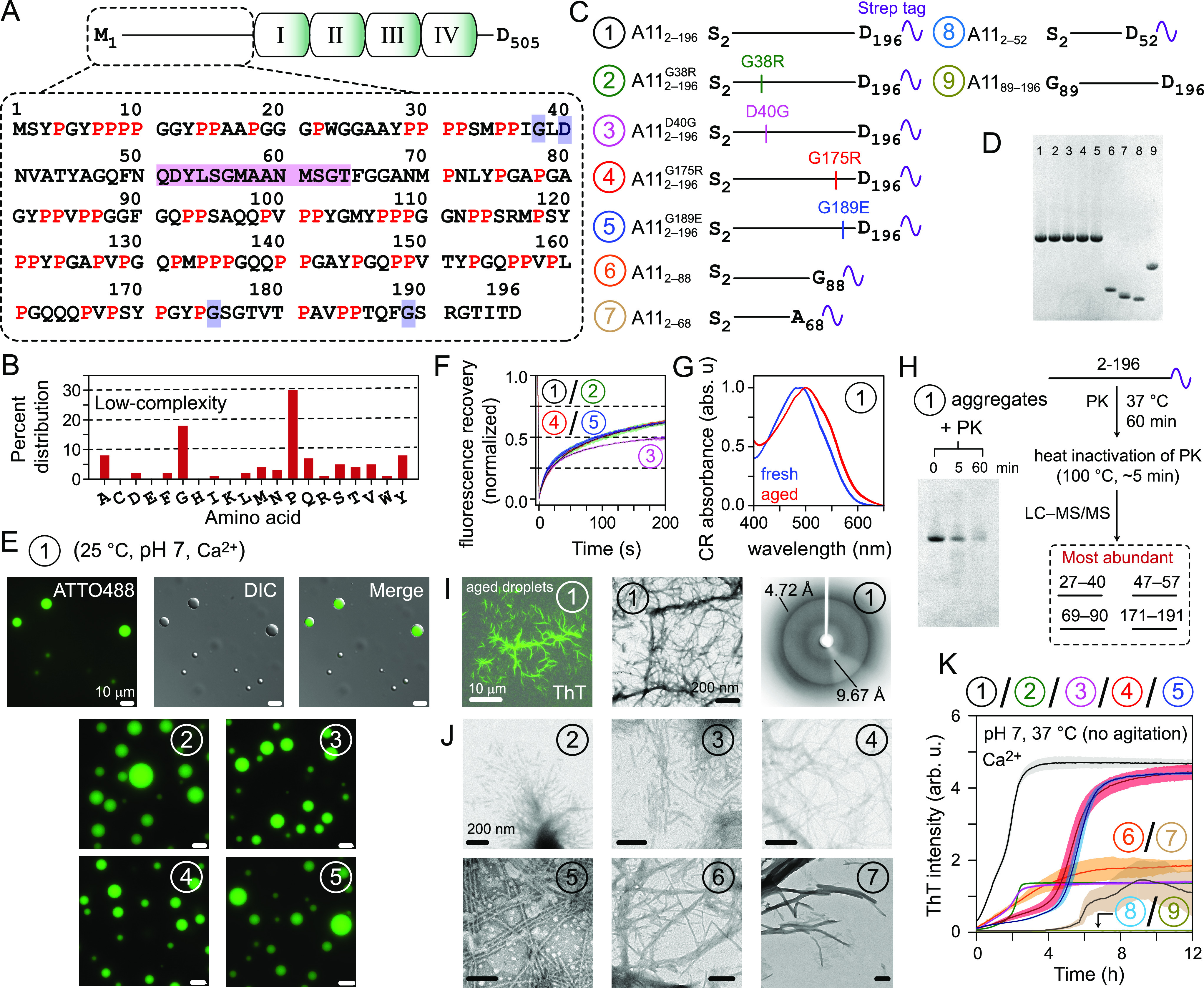
Aggregation of A11-PRD. (A) A11 organization and primary sequence of A11-PRD; I–IV (annexin repeats); prolines (red); locations of ALS-associated mutations (blue); proposed S100A6 binding site (magenta). (B) A11-PRD composition. (C) A11-PRD constructs tested in this study; each construct is designated by a circled number. (D) SDS-PAGE analysis of A11-PRD constructs; throughout the figure, the circled numbers signify the constructs shown in panel (C). (E) Microscopy images of droplets of ATTO488-labeled constructs 1–5 (50 μM each). (F) FRAP curves of droplets of constructs 1–5, *n* = 3; mean (solid line), SD (shaded region). (G) CR absorbance spectra of A11_2–196_ aggregates (red) and freshly prepared solutions (blue); *n* = 3. (H) SDS-PAGE analysis of PK digestion of A11_2–196_ aggregates (left) and LC–MS/MS scheme and results (right). (I) Fluorescence microscopy (left), TEM (middle), and X-ray diffraction (right) of A11_2–196_ fibrils. (J) TEM images of fibrils of constructs 2–7. (K) ThT assays of constructs 1–9 (50 μM), *n* = 3; mean (solid line), SD (shaded region).

The following recombinant constructs were tested in this study (Figures 1C,D and S1): (a) full-length A11-PRD, A11_2–196_, and its ALS variants, A11_2–196_^G38R^, A11_2–196_^D40G^, A11_2–196_^G175R^, and A11_2–196_^G189E^; and (b) truncated A11-PRD, A11_2–88_, A11_2–68_, A11_2–52_, and A11_89–196_; the subscripts signify A11 residues. Only full-length constructs spontaneously phase separated into spherical condensates (Figure 1E). The latter often coalesced and increased in size with time (Video S1). Fresh condensates of full-length constructs showed ∼70% fluorescence recovery after photobleaching (FRAP), except for A11_2–196_^D40G^, where recoveries were ∼50% (Figure 1F). These results are consistent with prior in vivo observations^2^ and indicate that the cycling between phase-separated and soluble states was comparatively hampered in A11_2–196_^D40G^. A11_2–196_ exhibited time-dependent precipitation, and spectral-shift assays performed using an amyloid-specific probe, Congo red (CR), showed shifts toward 540 nm, indicating amyloidogenic aggregates (Figure 1G).^7^ SDS-PAGE analysis of proteinase K (PK) digestion of A11_2–196_ aggregates showed that the A11_2–196_ band persisted after ∼60 min of incubation with PK (Figure 1H). Thus, A11_2–196_ aggregates were proteolysis-resistant, as expected for amyloids.^7^ LC–MS/MS analysis of limited PK digestion of A11_2–196_ aggregates revealed that fragments comprising residues 27–40, 47–57, 69–90, and 171–191 were abundant (Figure 1H), indicating their incorporation into the aggregate core. Fluorescence and transmission electron microscopy (TEM) analyses of A11_2–196_ aggregates revealed ribbon-like fibrils, while their powder X-ray diffraction showed two rings at 4.72 and 9.67 Å, establishing cross-β architecture (Figure 1I).^7^ Similar fibrils were observed for ALS variants and two truncated A11-PRD constructs, A11_2–88_ and A11_2–68_ (Figure 1J). Aggregation kinetics of A11_2–196_ fibrils monitored using an amyloid-sensitive dye, thioflavin T (ThT), exhibited sigmoidal profiles, a hallmark of fibril formation,^7^ with ∼1.6 h to reach half-maximal signal (*t*_1/2_; Figure 1K). ALS variants showed longer lag times but similar growth phases (*t*_1/2_: ∼2, ∼1.8, ∼5.4, and ∼5.6 h for A11_2–196_^G38R^, A11_2–196_^D40G^, A11_2–196_^G175R^, and A11_2–196_^G189E^, respectively), revealing two distinct clusters for these N- and C-terminal ALS variants. These mutants thus may undergo slower primary nucleation,^8^ possibly because residues 27–40 and 171–191 were incorporated into the fibril core and mutations in these segments are likely to affect nucleation events. Among the truncated constructs, A11_2–88_ and A11_2–68_ exhibited slower fibrillization (*t*_1/2_: ∼ 3 and ∼7 h, respectively), confirming the supporting role of residues 171–191 and 69–90 in A11-PRD fibrillization. The remaining truncated constructs were soluble.

To understand the slower aggregation of ALS variants of A11-PRD against abundant A11 aggregates observed in ALS patients,^6^ we investigated the contributions of S100A6 overexpressed in ALS.^6,9^ Although copelleting assays have implicated residues 49–62 of rabbit A11 (residues 51–64 of human A11; Figure 1A) in S100A6 binding,^4^ the interactions between human A11-PRD and S100A6 are relatively unexplored by quantitative biophysical techniques. Based on sedimentation, S100A6 was dimeric, consistent with a previous report.^10^ Sedimentation profiles of the A11_2–196_–S100A mixture revealed the formation of a 1:1 complex between the A11_2–196_ monomer and S100A6 dimer (Figure 2A). Surprisingly, excess A11_2–196_ in this mixture was monomeric and likely disordered (Table S1). Given that cloudy A11_2–196_ solutions cleared upon the addition of S100A6, this may alter the aggregation of A11_2–196_. Sedimentation measurements also revealed the formation of 1:1 complex between A11_2–88_/A11_2–68_/A11_2–52_ monomers and S100A6 dimer (Figure S2). Surface plasmon resonance (SPR) measurements using immobilized A11_2–196_ and S100A6 dimer as the binding analyte yielded a dissociation constant (*K*_D_) of 71 nM (Figure 2B and Tables 1 and S2; all reported *K*_D_ values are for an S100A6 dimer:A11-PRD monomer stoichiometry). The ALS variants exhibited similar *K*_D_ values ranging between 33–51 nM, establishing that ALS-linked mutations did not significantly affect interactions between A11-PRD and S100A6 (Table 1 and Figure S3). Additionally, the truncated constructs, A11_2–88_ and A11_2–68_, yielded similar *K*_D_ values (43 and 121 nM, respectively), whereas A11_2–52_–S100A6 interactions were ∼120-fold weaker by isothermal titration calorimetry (ITC, 6 μM; Table 1 and Figure S4). A11_89–196_ did not bind to S100A6. Therefore, we conclude that S100A6 binds to a high-affinity site between residues 52–68 and a weak-affinity site between residues 2–52 of A11-PRD. Consequently, ALS-associated mutations do not significantly affect A11-PRD–S100A6 interactions, as these are located away from the high-affinity binding site.

**Table 1:** Quantitative Analysis of A11-PRD–S100A6 Interactions^a^

full-length A11-PRD	*K*_D_ (nM)	truncated A11-PRD	*K*_D_ (nM)
A11_2–196_	71 ± 4	A11_2–88_	43 ± 5
A11_2–196_^G38R^	33 ± 5	A11_2–68_	121 ± 23
A11_2–196_^D40G^	38 ± 4	A11_2–52_	6000 ± 400^b^
A11_2–196_^G175R^	51 ± 9	A11_89–196_	–c
A11_2–196_^G189E^	38 ± 14		

a Affinities were measured using SPR (*n* ≥ 2), unless noted otherwise, and refer to an S100A6 dimer:A11-PRD monomer stoichiometry uncovered by sedimentation.

b Measured using ITC (*n* = 3, Figure S4).

c No binding was detected via sedimentation, SPR, and NMR.

**Figure 2:**
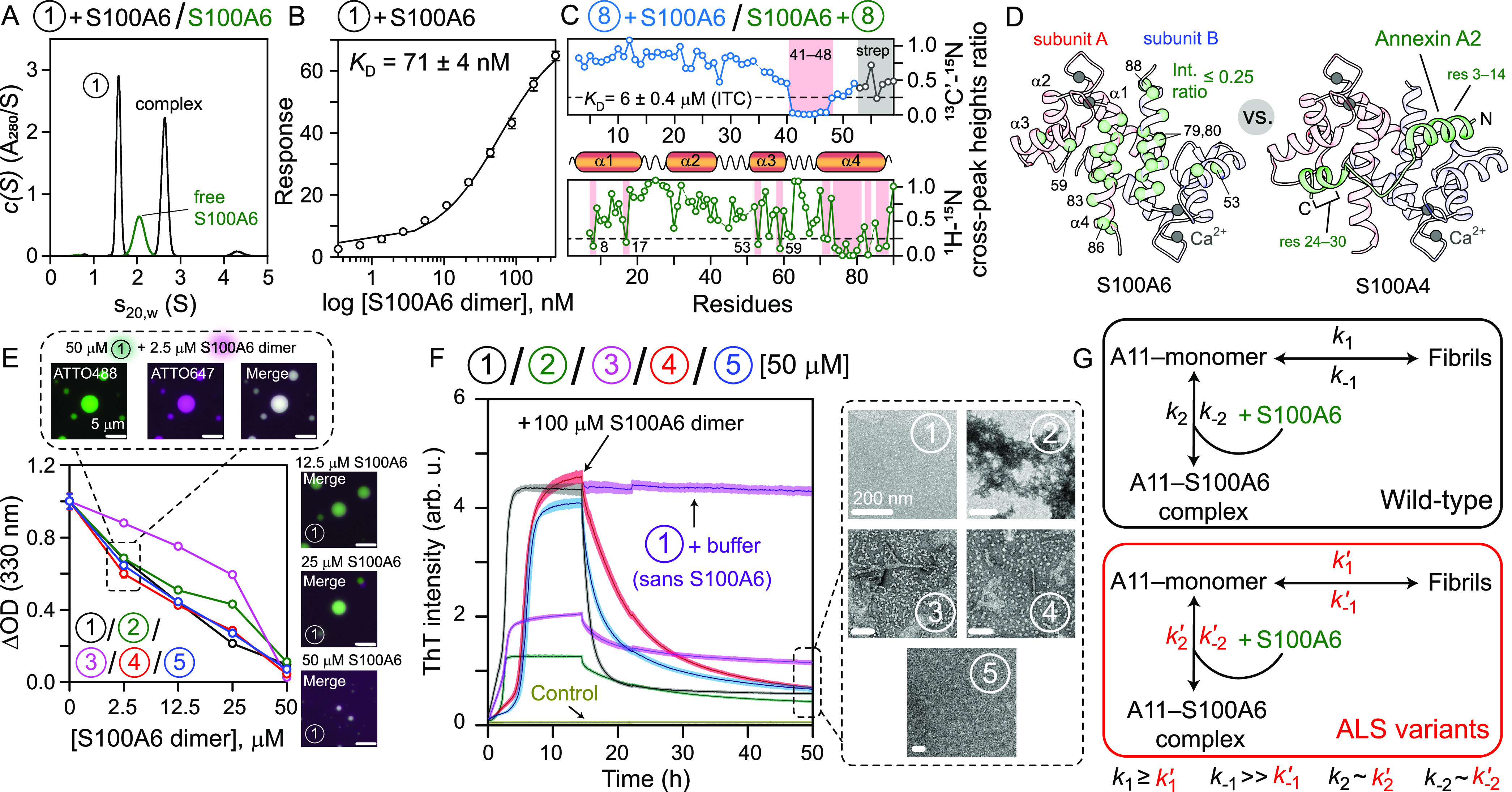
Impact of S100A6 on A11-PRD aggregation. (A) Sedimentation profiles of S100A6 (green) and S100A6 + A11_2–196_ (same numbering scheme as Figure 1C) mixture, black. (B) SPR analysis of A11_2–196_ – S100A6 interactions; *n* = 3, data (circles), fit (solid line). The reduction in cross-peak heights of (C, upper) 200 μM ^13^C/^15^N-labeled A11_2–52_ (blue) with 25 μM S100A6 dimer and (C, lower) 100 μM ^15^N-labeled S100A6 dimer with 50 μM A11_2–52_ (green). X-ray structures of (D, left) S100A6;^12^ affected residues in panel (C) are shown as green spheres, and (D, right) S100A4–Annexin A2 complex;^13^ residues 33–339 of A2 are not shown for clarity. (E) Dissolution of ATTO488-labeled condensates of constructs 1–5 with ATTO647-labeled S100A6 monitored using turbidity assays and fluorescence microscopy. (F) S100A6’s impact on fibrils of constructs 1–5. Each construct (50 μM, *n* = 3) was incubated for ∼14 h, upon which 100 μM S100A6 dimer was added; TEM images of samples at 50 h are shown (right). Additional three replicates of each construct received buffer at ∼14 h; only A11_2–196_ after the addition of buffer is shown (purple). Additional controls included each construct (50 μM, *n* = 3) with 100 μM S100A6 dimer at 0 h; only A11_2–196_ is shown (olive). (G) Model describing the findings of this study.

To obtain residue-specific details about the above-described weak-affinity site, we performed nuclear magnetic resonance (NMR) backbone assignments of S100A6 and A11_2–52_. Unlike A11_2–52_, amyloidogenic A11-PRD constructs are refractory to these measurements due to their fibrillization, and therefore, the corresponding assessment of the high-affinity site is not plausible. NMR chemical shift analysis^11^ revealed that A11_2–52_ was disordered in solution (Table S3). NMR titration experiments found significant attenuation in the ^13^C’-^15^N cross-peaks of residues ^41^NVATYAGQ^48^ of A11_2–52_ upon the addition of S100A6 (Figures 2C and S5), establishing that residues 41–48 of A11-PRD represent the weak-affinity binding site for S100A6. Significant attenuation in multiple ^1^H–^15^N cross-peaks of the S100A6 dimer was observed in the presence of A11_2–52_ (Figures 2C and S6). Figure 2D maps these perturbations onto the S100A6 structure,^12^ which showed that the affected residues lie in and around the dimerization interface. The Annexin A2–S100A4 complex (other members of the Annexin- and S100-families) is shown in Figure 2D,^13^ where the A2 head domain binds to both subunits of the S100A4 dimer. Given the similarities between this complex and our results, we conclude that A11-PRD likely binds to the S100A6 dimer asymmetrically.

The impact of S100A6 on condensates of A11_2–196_ was monitored by fluorescence microscopy and turbidity assays (Figure 2E). S100A6 readily colocalized with A11_2–196_ condensates, and its increasing concentrations led to their progressive decrease, establishing S100A6-mediated dissolution of these condensates. ALS variants showed similar results, although A11_2–196_^D40G^ showed a smaller decrease in turbidity at lower S100A6 concentrations. To determine the impact of S100A6 on A11-PRD fibrils, 2 mol equiv of S100A6 dimer were added to A11_2–196_ fibrils at a stationary phase. A significant loss of ThT signal was observed with *t*_1/2_: ∼3.5 h (Figure 2F). TEM images showed a complete lack of fibrils, establishing that the A11-PRD fibrillization can be reversed by S100A6. Although a similar decrease in ThT signals was noted for ALS variants, the corresponding *t*_1/2_ were longer (4–13 h). TEM images of A11_2–196_^G189E^ showed no aggregates, while amorphous (nonfibrillar) aggregates were seen in the case of A11_2–196_^G38R^. For the A11_2–196_^D40G^ variant, ThT signals did not return to baseline, indicating incomplete fibril dissolution, which was confirmed by TEM. Residual fibrillar aggregates were also observed for A11_2–196_^G175R^, which underwent the slowest dissolution (*t*_1/2_ ∼ 13 h).

Figure 2G depicts a model that is consistent with these results. A11-PRD and ALS variants have similar binding affinities and, thus, likely similar exchange kinetics for S100A6. The latter can sequester monomeric A11-PRD in a soluble form, inhibiting its fibrillization. Because of longer half-times but similar growth phases, A11-PRD and its ALS variants may have equivalent forward rate constants for monomer-to-fibril transitions. However, the corresponding fibril-to-monomer rate constant is likely much faster in A11-PRD than its ALS variants, increasing the amount of available monomer, which can be siphoned off by S100A6, leading to fibril dissolution. In contrast, ALS variants would produce fewer free monomers from fibrils over time, resulting in slower S100A6-mediated fibril dissolution and possibly leading to abundant neuronal A11 inclusions. Given that S100-family proteins, including S100A6, can also disassemble nonmuscle myosin-2 filaments,^14^ depolymerization is likely one of their many functions, providing a plausible explanation as to why S100A6 is overexpressed in ALS. Finally, we note that PRDs are ubiquitous in eukaryotes and often form dynamic signaling networks due to their disordered conformations and favorable binding properties.^15^ The results presented in this study demonstrate that some PRDs can also phase separate and form labile amyloid fibrils that dissolve upon interaction with partner proteins. Thus, while the structural basis of how a PRD forms β-sheet–rich fibrils is not known and is a topic of ongoing research in our laboratory, we argue that A11-PRD may represent a new subclass of PRDs that are prone to reversible aggregation.

## Methods

### Protein Expression and Purification

A11-PRD and S100A6 constructs were custom synthesized (Azenta Life Sciences) and expressed at 16 °C. Tobacco etch virus (TEV) protease construct, a generous gift from David Waugh (NIH), was expressed at 37 °C. For A11-PRD and S100A6, cells were grown at 37 °C in 1 L Luria–Bertani or minimal M9^15−17^ medium. The latter was used for NMR isotopic labeling. TEV purification was described previously.^17^ A11-PRD constructs, except A11_89–196_, were purified as follows: Cells, resuspended in a lysis buffer comprising 50 mM Tris, pH 8, and 6 M guanidine hydrochloride (GdmCl), were lysed by heat shock (80 °C for 5 min) and cleared by centrifugation. The supernatant was filtered (Stericup, Sigma-Aldrich) before being loaded onto a HisTrap column (Cytiva) pre-equilibrated with lysis buffer. Bound protein was washed with a buffer comprising 50 mM Tris, pH 8.0, and 250 mM NaCl and eluted in the same buffer containing 500 mM imidazole. The eluted protein was loaded onto a XK 16/20 column (Cytiva) prepacked with Strep-Tactin Sepharose resin (Cytiva) pre-equilibrated with 50 mM Tris, pH 8.0, 250 mM NaCl, 1 mM DTT, and 1 mM EDTA, and eluted in the same buffer containing 2.5 mM d-desthiobiotin. The protein was then mixed with TEV to hydrolyze the N-terminal B1 domain of protein G (GB1)^18^ fusion tag. The reaction was carried out at room temperature (∼20 h) and produced a poorly soluble hydrolyzed product. The latter was solubilized by using 6 M GdmCl and passed through a HisTrap column. Relevant flow-through fractions were purified using reverse-phase HPLC (Jupiter 10 μm C18 300 Å column) with a 25–37% acetonitrile gradient comprising 0.1% trifluoroacetic acid (TFA). The eluted protein was lyophilized and stored at −80 °C. For A11_89–196_, similar lysis and HisTrap purification steps were performed. The eluted protein was then purified using a HiLoad 26/600 Superdex 75 prep-grade column (Cytiva) pre-equilibrated with 50 mM Tris, pH 8.0, 250 mM NaCl, and 1 mM EDTA. Relevant fractions were incubated with TEV to cleave off the GB1 tag. The hydrolyzed product was passed through a HisTrap column and further purified by reversed-phase HPLC. Eluted fractions were lyophilized and stored at −80 °C.

For S100A6, cells were resuspended in a lysis buffer containing 50 mM Tris, pH 8.0, and 2 mM CaCl_2_, lysed using EmulsiFlex-C3 (Avestin), and cleared by centrifugation. The supernatant was loaded onto a XK 16/20 column prepacked with Q-Sepharose HP resin (Cytiva) pre-equilibrated in lysis buffer and eluted with a 0–1 M NaCl gradient in the same buffer. Eluted fractions were loaded onto a XK 16/20 column prepacked with Phenyl Sepharose HP resin (Cytiva) pre-equilibrated in 50 mM Tris, pH 8.0, and 2 mM CaCl_2_, and eluted using the same buffer (sans CaCl_2_) containing 5 mM EGTA. The eluted protein was purified using a HiLoad 26/600 Superdex 75 prep grade column (Cytiva) pre-equilibrated in 50 mM Tris, pH 8.0, and 2 mM CaCl_2_. Relevant fractions were dialyzed against 25 mM HEPES, pH 7.0, and 5 mM CaCl_2_, and stored at −80 °C.

### Phase Separation and Turbidity Measurements

Lyophilized A11-PRD constructs were reconstituted in DMSO, followed by immediate dilution in a buffer comprising 25 mM HEPES, pH 7.0, and 5 mM CaCl_2_ (final DMSO concentration ∼ 2% v/v). For A11_2–196_ + S100A6 mixtures, A11_2–196_ condensates were made, followed by the addition of different concentrations of the S100A6 dimer. These mixtures were analyzed after an ∼5 min incubation period. Turbidity was recorded at optical densities (OD) of 330 and 600 nm using an Agilent Cary 50 Bio UV–vis spectrophotometer. The values reported in Figure 2E are relative to those of the corresponding A11-PRD samples without S100A6.

### Fluorophore Labeling

A11-PRD constructs were mixed with a 4 mol equivalent of ATTO-488 NHS ester in 25 mM HEPES, pH 8, and 20% v/v DMSO. The reaction was performed at room temperature (30 min) before being quenched by dilution into a buffer comprising 50 mM Tris, pH 8, and 6 M GdmCl. Constructs were further purified using reversed-phase HPLC, mixed with corresponding unconjugated proteins (concentration of fluorophore-labeled protein = 5 mol %), lyophilized, and stored at −80 °C.

S100A6 was mixed with 4 mol equiv of ATTO-647N maleimide in 25 mM HEPES, pH 7, and 5 mM CaCl_2_. The reaction was performed at room temperature (30 min) and quenched by β-mercaptoethanol (BME). Excess dye and BME were removed by dialysis. The fluorophore-labeled S100A6 was mixed with unconjugated protein (concentration of fluorophore-labeled protein = 5 mol %) and stored at −80 °C.

### Microscopy Imaging and FRAP Assays

For microscopy experiments, slides were passivated using PEG-silane.^19,20^ Differential interference contrast (DIC) imaging was performed on a Nikon Ti2 widefield microscope equipped with a DS-Qi2 CMOS camera and 100*×*/1.49NA oil DIC N2 Objective as described previously.^29^ Condensates of A11-PRD constructs were excited by a 488 nm laser controlled by a Lumencor SpectraX instrument for imaging of ATTO-488. Additionally, a 640 nm laser was used to image the ATTO-647N-labeled S100A6. For the image shown in Figure 1I, droplets of 50 μM A11_2–196_ with 20 μM ThT were incubated at 37 °C for 14 h, and image was taken using a 488 nm laser. FRAP measurements of ATTO-488-labeled A11-PRD constructs were performed on a Nikon point scanning confocal C2 with 2 GaAsP PMTs using a Plan Apo λ 100*×*/1.45 NA oil objective using our previously described procedure.^29^

### CR Assay, PK Digestion, and TEM

CR assay, PK digestion, and TEM measurements were carried out using our previously described protocols.^16,21^

### X-ray Diffraction

A11_2–196_ (50 μM) was incubated for 3 days (37 °C) and subsequent fibrils were pelleted using Optima XE Ultracentrifuge (Beckman Coulter). A small amount of the sample was dried and loaded onto a Cryoloop. The sample was mounted on a Bruker Microstar 592 diffractometer equipped with an APEX II CCD detector and Cu Kα radiation (λ = 1.54178 Å). X-ray data was collected using a 360° scan with an exposure time of 300 s.

### Fibril Formation and Dissolution Kinetics

Measurements were performed at 37 °C under nonagitated conditions using a microplate reader (Infinite M Plex; Tecan) and sealed 96-well flat bottom plates containing 100 μL of sample per well. 50 μM samples of individual A11-PRD constructs were prepared as described above. To ensure reproducible results, the concentrations of stock solutions in DMSO and those of the final samples were checked by UV absorbance at 280 nm. Fibrillization was monitored using ThT (20 μM) fluorescence collected every 5 min (excitation and emission wavelengths: 415 and 480 nm, respectively). Because the aggregation was dependent on the time required to plate different samples, we used two batches to determine rates. Batch 1 comprised A11_2–196_, A11_2–196_^G38R^, A11_2–196_^D40G^, A11_2–196_^G175R^, and A11_2–196_^G189E^ (*n* = 2 × 3; see below), while batch 2 comprised A11_2–196_, A11_2–88_, A11_2–68_, A11_2–52_, and A11_89–196_ (*n* = 3). In both cases, A11_2–196_ aggregation was used to benchmark the results. Additionally, batch 1 comprised three replicates of 50 μM A11_2–196_/A11_2–196_^G38R^/A11_2–196_^D40G^/A11_2–196_^G175R^/A11_2–196_^G189E^ + 100 μM S100A6 dimer mixtures. These mixtures did not show any increase in ThT fluorescence, indicating a lack of fibrillization. Similarly, A11_2–52_ and A11_89–196_ (batch 2) did not form fibrils. To monitor S100A6-mediated dissolution of A11-PRD fibrils, samples from batch 1 (see above) were allowed to aggregate to reach a ThT signal plateau (total incubation time ∼14 h). 100 μM S100A6 dimer was then added to three replicates of each construct, namely, A11_2–196_, A11_2–196_^G38R^, A11_2–196_^D40G^, A11_2–196_^G175R^, and A11_2–196_^G189E^. Additionally, negative controls of three replicates received only buffer (sans S100A6). S100A6-dissolution of fibrils was assessed using the drop in ThT signal. Samples of negative controls did not show any noticeable decrease in ThT signal.

### SPR

Measurements were performed in 25 mM HEPES, pH 7.0, 5 mM CaCl_2_, and 0.05% surfactant Polysorbate 20 (Cytiva) at 25 °C. All A11-PRD constructs were immobilized on a Series S Sensor Chip CM5 (Cytiva), except A11_2–88_, which was immobilized on a Series S Sensor Chip CM7 (Cytiva). Due to the relative ease of immobilization, measurements were carried out with GB1-tagged A11-PRD constructs.

Multicycle kinetic experiments were performed with S100A6 dimer as the binding analyte. Concentrations ranged from 700 pM to 5 μM (in monomeric subunits), with association times of 600–720 s and dissociation times of 60–180 s at a flow rate of 20 μL/min. The immobilization surface was regenerated with 6 M GdmCl for 30 s at a flow rate of 30 μL/min. Each experiment was performed in triplicate. However, due to significant aggregation observed with A11_2–196_^G175R^ and A11_2–196_^G189E^, only two replicates of these two constructs were analyzed. The following equation was used to determine *K*_D_.
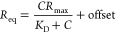
Iwhere *R*_eq_ is the steady state binding level and *C* is the analyte concentration. *R*_max_ (analyte binding capacity of the surface, in response units), offset (residual response at zero concentration, in response units), and *K*_D_ (in M) are fitted parameters.

The initial experiments were performed with S100A6 concentrations ranging from 10 pM to 10 μM (in monomeric subunits). But we were unable to characterize the low-affinity interaction between A11_2–52_ and S100A6, and no binding was detected for A11_89–196_.

### Sedimentation Velocity Analytical Ultracentrifugation

Sedimentation experiments were carried out at 50,000 rpm and 20 °C on a Beckman Coulter ProteomeLab XL-I analytical ultracentrifuge and an An-50-Ti rotor following standard protocols.^22^ Stock solutions of individual constructs were diluted to ∼20 and ∼50 μM. Mixtures were prepared by mixing the components at appropriate volumes. Samples were spun at 12,000*g* for 2 min at room temperature to remove any precipitated proteins and then loaded into 12 mm two-channel centerpiece cells. Absorbance sedimentation data were collected at 280 nm and analyzed using our published protocols.^15,17,23−25^

### NMR

NMR samples were prepared in a buffer comprising 25 mM HEPES, pH 7.0, 5 mM CaCl_2_, 1 mM TCEP, and 7% (v/v) D_2_O. Experiments were carried out at 30 °C on Bruker 600 and 800 MHz spectrometers equipped with z-gradient triple resonance cryoprobes. Spectra were processed using NMRPipe^26^ and analyzed using CCPN.^27^ Backbone resonance assignments of A11_2–52_ and S100A6 were carried out using TROSY-based triple resonance experiments and (HACA)N(CA)CON experiment.^28^ The latter was used for A11_2–52_ due to its high-proline content. NMR titration experiments (2D ^1^H_N_-^15^N TROSY-HSQC) were performed using the 0.1 mM ^15^N-labeled S100A6 dimer and 0.05 mM unlabeled A11_2–52_. Similar experiments were carried out using the 0.05 mM ^15^N-labeled S100A6 dimer and 0.3 mM unlabeled A11_89–196_ and established the lack of S100A6–A11_89–196_ interactions. Similar titration measurements were carried out using 0.2 mM ^15^N/^13^C-labeled A11_2–52_ and 0.025 mM unlabeled S100A6 dimer. Due to high proline content of A11_2–52_, measurements were also performed using 2D ^13^C–^15^N CON correlation experiments.^28^

### ITC

Measurements were performed using a low-volume Affinity ITC calorimeter (TA Instruments) at 25 °C in 25 mM HEPES, pH 7.0, and 5 mM CaCl_2_. 3.6–4.2 μL aliquots of 300 μM A11_2–52_ were injected (20 injections) into a cell containing 50 μM S100A6 dimer (*n* = 3). Results were analyzed by using NanoAnalyze software (TA Instruments).

## Data Availability

All A11-PRD and S100A6 plasmids reported in this study were deposited in the Addgene repository, https://www.addgene.org (accession no. 196208–196217). The NMR chemical-shift assignments of A11_2–52_ and S100A6 were deposited in the Biological Magnetic Resonance Bank, https://bmrb.io (entry no. 51795 and 51796, respectively). LC–MS/MS data were deposited in MassIVE repository, https://massive.ucsd.edu/ (Data set Identifier: MSV000091107).

## Supporting Information Available

A11-PRD constructs used in current study and AUC, SPR, ITC, and NMR analyses of their interactions with S100A6; parameters obtained from AUC and NMR and technical details of SPR measurements; caption for Video S1 (PDF)
Fusion of A11-PRD droplets (MP4)
