# Supplementary material for: ALS Variants of Annexin A11’s Proline-Rich Domain Impair Its S100A6-Mediated Fibril Dissolution
Source: ACS Chem Neurosci. 2023 Jul 11;14(15):2583–9. doi: 10.1021/acschemneuro.3c00169 (PMC10401653; doi:10.1021/acschemneuro.3c00169)
Supplement: Supplementary file 1 — cn3c00169_si_001.pdf [file cn3c00169_si_001.pdf]

Supporting Information

ALS Variants of Annexin A11's Proline-rich Domain Impair Its S100A6-mediated Fibril Dissolution.

Aman Shihora,[1§] Ruben D. Elias,[1§] John A. Hammond,[2] Rodolfo Ghirlando,[3] and Lalit Deshmukh[1,*]

[1]Department of Chemistry and Biochemistry, University of California San Diego, La Jolla, CA 92093, USA, [2]Scripps Research Biophysics and Biochemistry Core, The Scripps Research Institute, La Jolla, CA 92037, USA, [3]Laboratory of Molecular Biology, National Institute of Diabetes and Digestive and Kidney Diseases, National Institutes of Health, Bethesda, MD 20892, [§]Equal contribution.

[*]To whom correspondence should be addressed. Email: ldeshmukh@ucsd.edu

This PDF file includes:

Supplementary Figures S1 to S6

Supplementary Tables S1 to S3

Caption for Supplementary Video S1

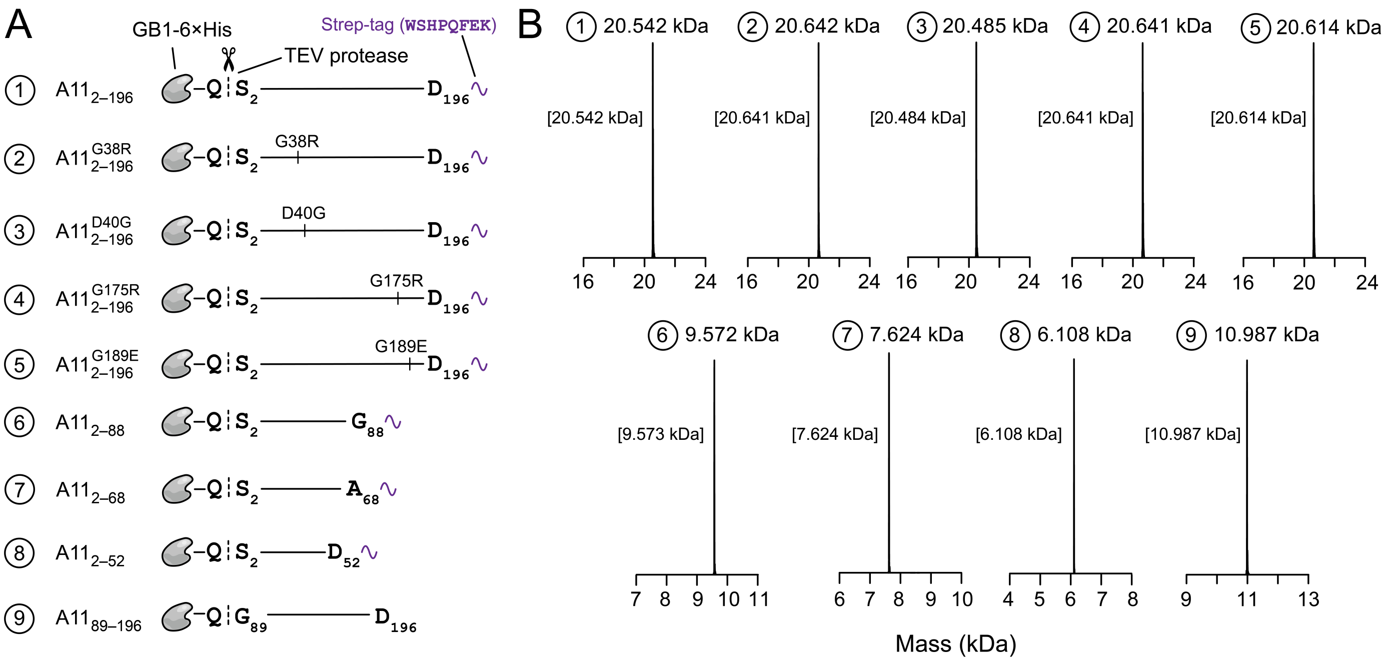

Figure S1. Recombinant A11-PRD constructs used in current study. (**A**) List of A11-PRD constructs. Each construct is designated by a circled number. The locations of the TEV cleavage sites are marked by vertical dashed lines and scissors. GB1-6xHis denotes the N-terminal B1 domain of protein G (GB1) tag, used to enhance the expression levels, followed by a spacer sequence and a polyhistidine (6xHis) affinity tag. Additionally, all A11-PRD constructs except for A11$_{89–196}$ (construct no. 9), carried a non-cleavable C-terminal Strep tag, magenta. Unlike A11-PRD constructs, S100A6 did not carry any fusion tags. All A11-PRD and S100A6 constructs were subcloned in BL21(DE3) cells (Agilent). (**B**) Analysis of TEV-cleaved recombinant proteins using liquid chromatography–electrospray ionization–time-of-flight mass spectrometry (LC–ESI–TOFMS); the numbers in parenthesis represent the corresponding theoretical masses.

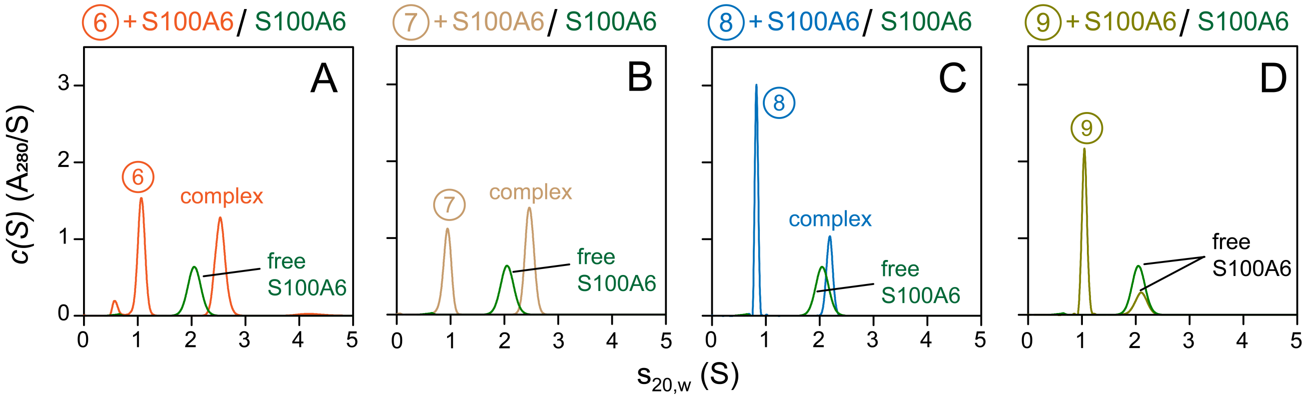

Figure S2. Analysis of interactions between truncated A11-PRD constructs and S100A6 by AUC. Absorbance sedimentation c(s) profiles of truncated A11-PRD constructs, namely (**A**) $A11_{2–88}$, (**B**) $A11_{2–68}$, (**C**) $A11_{2–52}$, and (**D**) $A11_{89–196}$ in the presence of S100A6; same numbering scheme as Fig. S1. Sedimentation profiles of free S100A6, which established the presence of a monodispersed dimeric species, are shown for reference (green). Addition of S100A6 dimer to $A11_{2–88}$, $A11_{2–68}$, and $A11_{2–52}$ resulted in a faster sedimenting complexes consistent with an S100A6 dimer:A11 monomer complex (**A**–**C**). In contrast, $A11_{89–196}$ did not bind to S100A6 (**D**). Additionally, sedimentation profiles of all excess truncated constructs revealed single monodispersed monomeric species. Loading protein concentrations were ~20 µM; in monomer units. All measurements were carried out at 20 ºC in 25 mM HEPES, pH 7, and 5 mM $CaCl_2$.

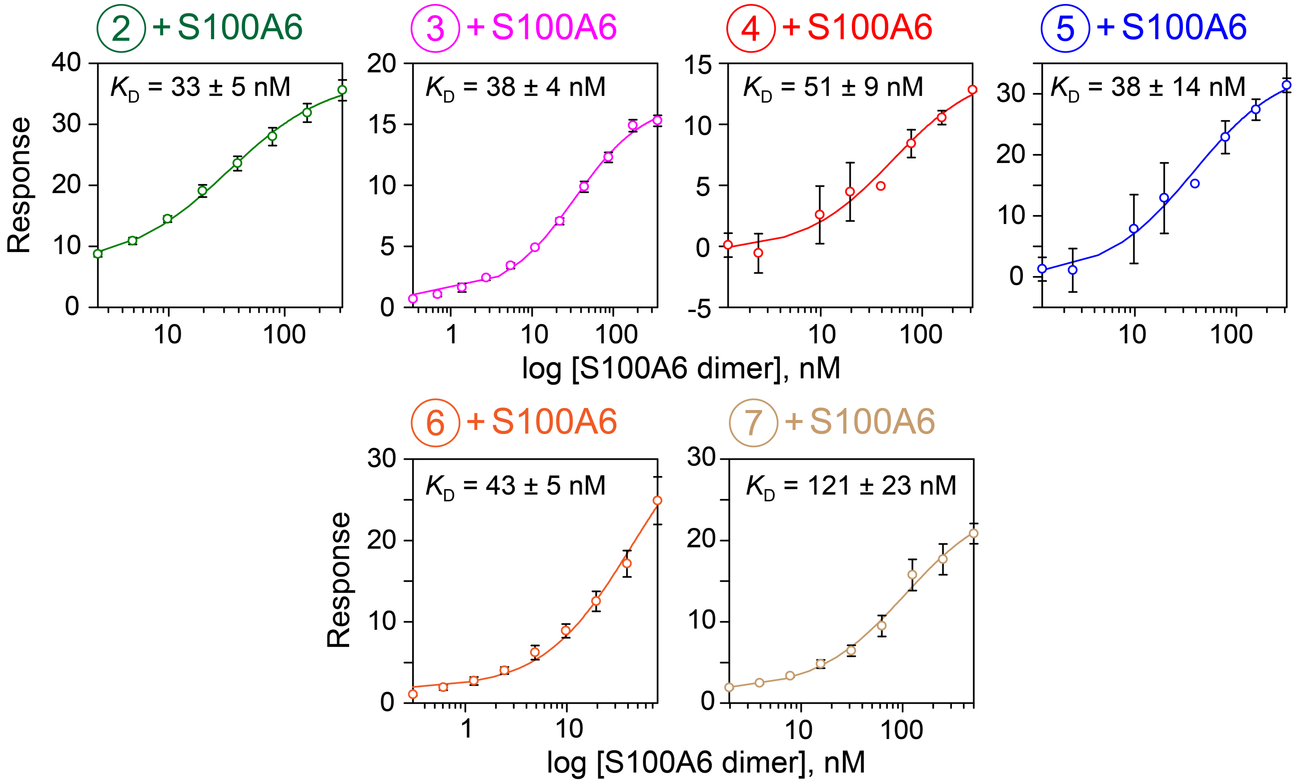

Figure S3. Analysis of interactions between A11-PRD constructs and S100A6 by SPR. Binding of A11-PRD constructs, namely $A11^{G38R}_{2\text{–}196}$, $A11^{D40G}_{2\text{–}196}$, $A11^{G175R}_{2\text{–}196}$, $A11^{G189E}_{2\text{–}196}$, $A11_{2\text{–}88}$, and $A11_{2\text{–}68}$ (construct no. 2 – 7; same numbering scheme as Fig. S1), to S100A6 is quantified by SPR spectroscopy. The experimental data are displayed as circles, and the solid lines represent individual fits to equation I (Methods, Main text); Error bars, S.D. values ($n \geq 2$). All measurements were carried out at 25 ºC in 25 mM HEPES, pH 7, and 5 mM $CaCl_2$.

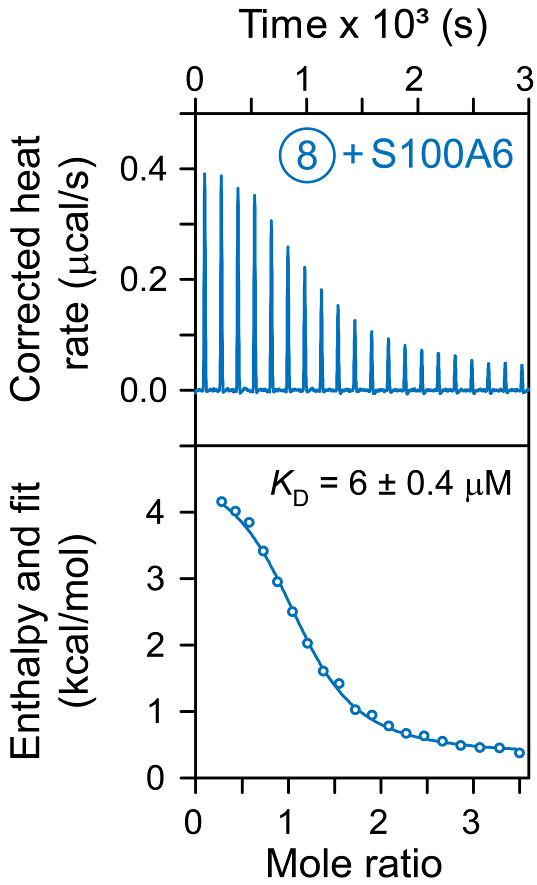

Figure S4. ITC analysis of S100A6 – A11$_{2\text{-}52}$ interactions. 300 µM A11$_{2\text{-}52}$ was titrated into 50 µM S100A6 dimer (*n* = 3). Isotherms were fitted to an independent-sites binding model in the software NanoAnalyze, TA Instruments (S100A6 dimer:A11-PRD monomer complex); Enthalpy (ΔH) = 4.2 ± 0.1 kcal/mol, Entropy (-TΔS) = -11.4 ± 0.1 kcal/mol. All measurements were performed at 25 ºC in 25 mM HEPES, pH 7, and 5 mM $CaCl_2$.

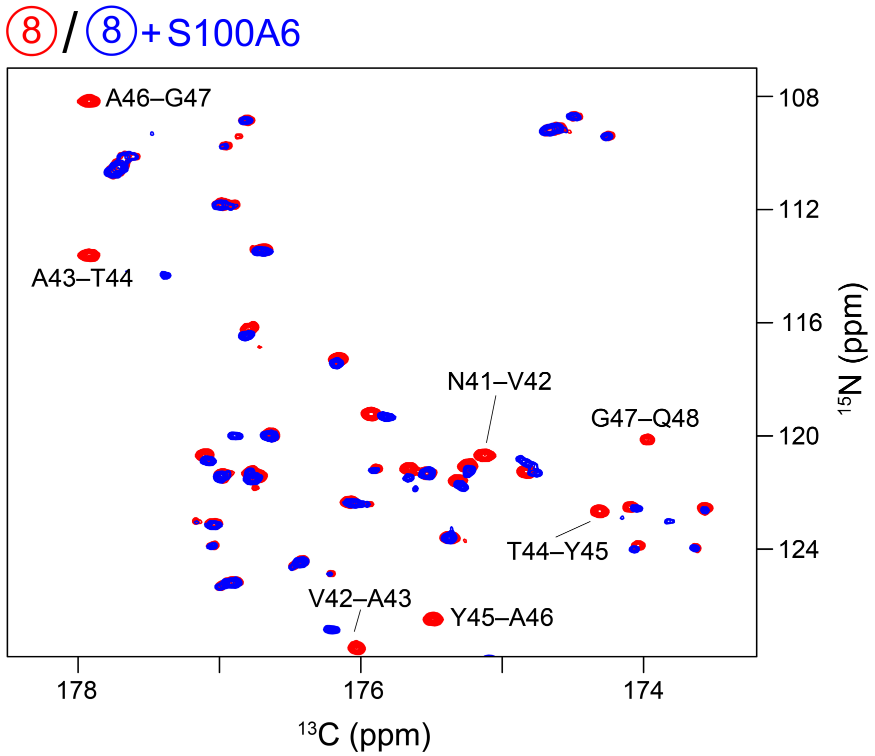

Figure S5. NMR analysis of A11$_{2\text{-}52}$ – S100A6 interactions. Overlay of the expanded region of the ^{13}C-^{15}N CON correlation spectra of ^{15}N/^{13}C-labeled 200 μM A11$_{2\text{-}52}$ (construct no. 8; same numbering scheme as Fig. S1) in the absence (red) and presence (blue) of 25 μM unlabeled S100A6 dimer. A few of the isolated cross-peaks of A11$_{2\text{-}52}$ that undergo significant signal attenuation (^{1}H-^{15}N cross-peak heights ratio < 0.25, cf. Fig. 2C, main text) due to the intermediate exchange upon addition of S100A6 dimer are labeled. Spectra were acquired at a spectrometer ^{1}H frequency of 800 MHz. Buffer and experimental conditions were 25 mM HEPES, pH 7, 1 mM TCEP, 5 mM $CaCl_2$, and 30 ºC.

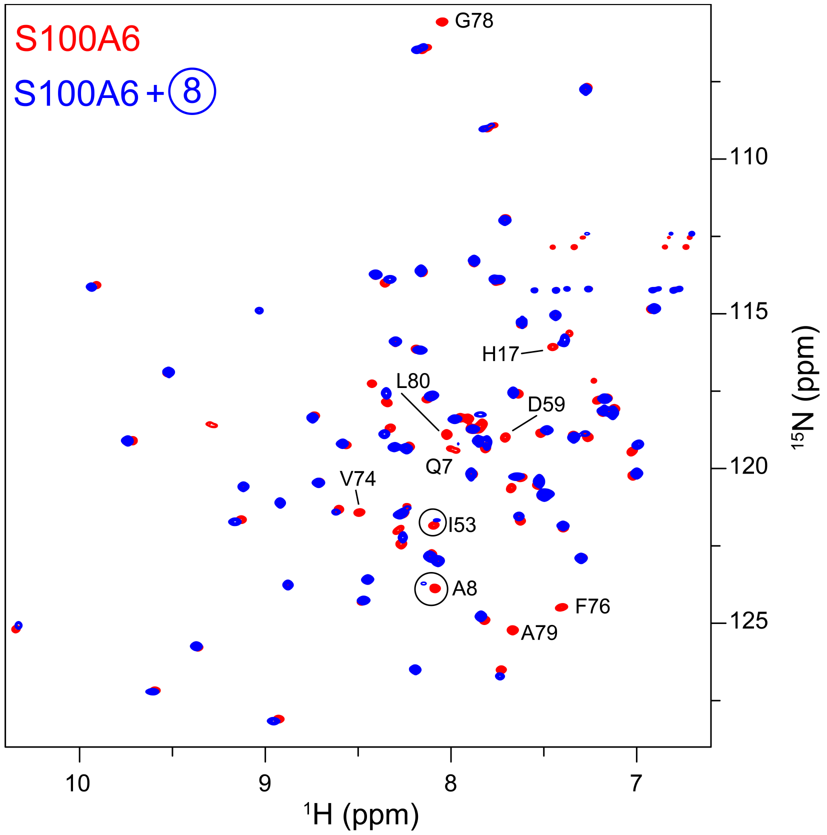

Figure S6. NMR analysis of S100A6 – A11$_{2\text{-}52}$ interactions. Overlay of the expanded region of the ^{1}H-^{15}N TROSY-HSQC correlation spectra of ^{15}N-labeled 100 µM S100A6 dimer in the absence (red) and presence (blue) of 50 µM unlabeled A11$_{2\text{-}52}$ (construct no. 8; same numbering scheme as Fig. S1). A few of the isolated cross-peaks of S100A6 that undergo significant signal attenuation (^{1}H-^{15}N cross-peak heights ratio < 0.25, cf. Fig. 2C, main text) due to the intermediate exchange upon addition of A11$_{2\text{-}52}$ are labeled. Spectra were acquired at a spectrometer ^{1}H frequency of 800 MHz. Buffer and experimental conditions were same as described in Fig. S5 caption.

Table S1. Best-fit frictional ratios obtained from sedimentation velocity measurements.[a]

Construct	Best-fit frictional ratio
$A11_{2–196}$	1.74
$A11_{2–88}$	1.66
$A11_{2–68}$	1.59
$A11_{2–52}$	1.36
$A11_{89–196}$	1.74
S100A6	1.22

[a] The best-fit frictional ratios of S100A6 indicate a monodispersed dimer, while those of A11-PRD constructs are indicative of disordered peptides.

Table S2. Immobilization levels for SPR measurements reported in this study.

Construct	Immobilization levels (Response units)
$A11_{2–196}$	152
$A11^{G38R}_{2–196}$	92
$A11^{D40G}_{2–196}$	38
$A11^{G175R}_{2–196}$	73
$A11^{G189E}_{2–196}$	123
$A11_{2–88}$	324
$A11_{2–68}$	393
$A11_{2–52}$	58
$A11_{89–196}$	127

Table S3. Residue-specific secondary structure populations of A11$_{2–52}$ derived from the backbone chemical shifts using δ2D.[a]

Residue no.	Amino acid	α	β	Coil	PPII	Secondary structure
2	S	—	—	—	—	—
3	Y	—	—	—	—	—
4	P	3.3	8.5	56.3	31.9	Coil
5	G	4.3	11.9	49.4	34.4	Coil
6	Y	3.2	13.5	46.1	37.1	Coil
7	P	—	—	—	—	—
8	P	—	—	—	—	—
9	P	—	—	—	—	—
10	P	3.7	13.4	56.6	26.3	Coil
11	G	4.4	15.3	53.6	26.7	Coil
12	G	3.6	15.7	53.9	26.8	Coil
13	Y	2.0	13.2	57.6	27.2	Coil
14	P	—	—	—	—	—
15	P	0.1	11.3	57.5	31.2	Coil
16	A	0.0	7.8	60.1	32.1	Coil
17	A	0.5	8.0	59.8	31.8	Coil
18	P	—	—	—	—	—
19	G	2.4	22.0	47.8	27.8	Coil
20	G	1.7	23.1	49.8	25.4	Coil
21	G	1.7	19.8	57.6	20.9	Coil
22	P	—	—	—	—	—
23	W	5.4	4.9	82.1	7.5	Coil
24	G	4.8	3.9	80.7	10.7	Coil
25	G	4.0	2.6	77.3	16.2	Coil
26	A	3.2	2.3	72.8	21.8	Coil
27	A	1.9	3.9	66.5	27.7	Coil
28	Y	0.9	7.9	59.0	32.2	Coil
29	P	—	—	—	—	—
30	P	—	—	—	—	—
31	P	—	—	—	—	—
32	P	0.1	19.9	50.5	29.4	Coil
33	S	0.1	27.9	45.2	26.8	Coil
34	M	0.1	39.6	35.1	25.2	Ext. strand

Table S3 (cont'd).

Residue no.	Amino acid	α	β	Coil	PPII	Secondary structure
35	P	—	—	—	—	—
36	P	2.4	22.7	51.4	23.4	Coil
37	I	4.0	11.5	61.7	22.8	Coil
38	G	3.9	7.7	64.6	23.7	Coil
39	L	3.9	4.8	68.4	22.9	Coil
40	D	2.6	4.1	70.8	22.5	Coil
41	N	1.8	2.8	74.2	21.3	Coil
42	V	1.2	2.7	74.1	22.0	Coil
43	A	1.4	3.7	72.0	23.0	Coil
44	T	2.0	5.3	68.9	23.7	Coil
45	Y	2.9	3.9	69.5	23.6	Coil
46	A	3.3	2.2	70.4	24.1	Coil
47	G	4.3	1.5	72.3	21.9	Coil
48	Q	5.0	1.5	71.7	21.8	Coil
49	F	6.0	1.4	72.1	20.5	Coil
50	N	—	—	—	—	—
51	Q	1.7	0.3	86.0	12.0	Coil
52	D	—	—	—	—	—

[a] The average secondary-structure propensities of $A11_{2–52}$ were as follows: ~2.6% of α-helix, ~10.1% of extended-β-sheet, and ~24.5% of the polyproline type II (PPII) helix; note that chemical shifts of the C-terminal strep tag residues were not considered in this assessment.

Video S1. Fusion of A11-PRD droplets. Montage of the fusion of phase-separated ATTO488-labeled $A11_{2-196}$ as a function of time. Fluorescence microscopy images were taken at room temperature in 25 mM HEPES, pH 7.0, and 5 mM $CaCl_2$ with 50 μM protein; scale bar = 5 μm.
